# *Tubaramure*, a Food-Assisted Integrated Health and Nutrition Program, Reduces Child Wasting in Burundi: A Cluster-Randomized Controlled Intervention Trial

**DOI:** 10.1093/jn/nxaa330

**Published:** 2020-11-26

**Authors:** Jef L Leroy, Deanna K Olney, Noé Nduwabike, Marie T Ruel

**Affiliations:** International Food Policy Research Institute, Washington, DC, USA; International Food Policy Research Institute, Washington, DC, USA; Institut de Statistiques et d'Études Économiques du Burundi, Bujumbura, Burundi; International Food Policy Research Institute, Washington, DC, USA

**Keywords:** wasting, program evaluation, effectiveness, infant and child, nutrition, COVID-19

## Abstract

**Background:**

Little is known about the impact of food-assisted maternal and child health programs (FA-MCHN) on child wasting.

**Objectives:**

We assessed the impact of *Tubaramure*, a FA-MCHN program in Burundi, on child (0 to 24 months) wasting and the differential impacts by socio-economic characteristics and age. The program targeted women and their children during the first 1000 days and included *1*) food rations, *2*) strengthening and promotion of use of health services, and *3*) behavior change communication (BCC).

**Methods:**

We conducted a 4-arm, cluster-randomized, controlled trial (2010–2012). Clusters were defined as “collines” (communities). Impact was estimated using repeated cross-sectional data (*n* = ∼2620 children in each round). Treatment arms received household and individual (mother or child in the first 1000 days) food rations (corn-soy blend and micronutrient-fortified vegetable oil) from pregnancy to 24 months (T24 arm), from pregnancy to 18 months (T18), or from birth to 24 months (TNFP). All beneficiaries received the same BCC for the first 1000 days. The control arm received no rations or BCC.

**Results:**

Wasting (weight-for-length *Z*-score <2 SD) increased from baseline to follow-up in the control group (from 6.5% to 8%), but *Tubaramure* had a significant (*P* < 0.05) protective effect on wasting [treatment arms combined, −3.3 percentage points (pp); T18, −4.5 pp] and on the weight-for-length *z*-score (treatment arms combined, +0.15; T24, +0.20; T18, +0.17). The effects were limited to children whose mother and household head had no education, and who lived in the poorest households. The largest effect was found in children 6 to 12 months of age: the group with the highest wasting prevalence.

**Conclusions:**

FA-MCHN programs in highly food-insecure regions can protect the most disadvantaged children from wasting. These findings are particularly relevant in the context of the economic crisis due to the coronavirus disease 2019 pandemic, which is expected to dramatically increase child wasting.

## Introduction

The evidence base on the effectiveness of nutrition-specific and nutrition-sensitive interventions to reduce the prevalence of child micronutrient deficiencies and linear growth faltering is rapidly expanding. Less is known, however, about effective approaches to prevent child wasting (1). Research on wasting has primarily focused on the treatment of moderate or severe acute malnutrition, especially in emergency contexts (1), a likely consequence of the high risks of mortality associated with these severe forms of wasting in children (2). Evidence on effective approaches to *prevent* wasting, however, is scant; the limited evidence suggests that interventions that provide food or nutrient supplements have a small, positive effect on weight-for-length in children 6–23 months of age (3).

The current coronavirus disease 2019 (COVID-19) pandemic is expected to lead to a dramatic increase in child wasting, mostly as a result of the global and national economic shocks it triggered (4). Based on recent projections, the number of children with wasting is estimated to increase by 6.7 million (from 47 to 53.6 million) during the first 12 months of the pandemic, with the largest burden found in sub-Saharan Africa and South Asia (5). Improving our understanding of how to prevent wasting is therefore more urgent than ever.

Food-assisted maternal and child health and nutrition (FA-MCHN) programs are a commonly used development strategy to address hunger, food insecurity, and undernutrition in low- and middle-income countries (6–8). FA-MCHN programs are an effective strategy to improve household food consumption and to reduce child linear growth faltering (6, 9, 10), but their impact on preventing child wasting is unknown. This paper presents findings from an evaluation of the FA-MCHN *Tubaramure* program, which had 3 core components: the distribution of food rations; activities to improve the provision of health services and to promote their use; and a behavior change communication (BCC) strategy focused on improving nutrition, health, and hygiene practices. Previously, we showed that the program improved household food security and increased household energy and micronutrient consumption. The program also had a positive effect on maternal dietary diversity and increased the proportion of children 6–23.9 months consuming ≥4 food groups. The effects on many of the consumption and dietary outcomes were shown to be attributable to the food rations (11). *Tubaramure* reduced the prevalences of child stunting and anemia in mothers and children (the study's primary outcomes), and had a small positive impact on children's motor and language development (12–14).

The primary objective of the analyses presented in this paper was to estimate the program's impact on child wasting. To investigate whether some children benefited more than others, we estimated the impact on wasting by socio-economic characteristics. Since the age of peak wasting prevalence differs by region, we also assessed whether the program's impacts varied by child age (15).

## Methods

### Study population

*Tubaramure* was implemented in Burundi's eastern provinces of Cankuzo and Ruyigi. Burundi is among the poorest countries in the world (16). It ranks 184 (out of 188) on the Human Development Index, and its population has very low levels of formal education (17). The country's population has endured genocides and a long history of political unrest. About half of Burundi's national budget came from foreign aid at the time our trial was conducted (18). From 2010 to 2012, inflation of consumer prices increased from 6.4% to 18.0% (19).

In 2010, before the *Tubaramure* intervention started, around half of the study households suffered from hunger (11), and 60% of children 6 to 24 months of age in our study population and 28% of their mothers were found to be anemic (13). The prevalence of stunting in children 24 to 42 months of age at baseline was 65% (14).

### The ***Tubaramure*** program

*Tubaramure* was implemented by a consortium of nongovernmental organizations (Food for the Hungry, International Medical Corps, and Caritas Burundi) led by Catholic Relief Services. The program was funded by the US Agency for International Development's Office of Food for Peace. Women at or after the fourth month of gestation and mothers of children less than 6 months of age were eligible to enroll in the program. Women needed proof from a health center–conducted pregnancy test and the pregnancy needed to show. *Tubaramure* included 3 core components. The food component consisted of a monthly family and individual ration of corn-soy blend (CSB) and micronutrient-fortified vegetable oil (**Supplemental Tables 1** and **2**). The objective of the family ration was to increase household food security in terms of both quantity and quality; the objective of the individual ration, targeted at pregnant women and women up to 6 months postpartum and at children starting at 6 months of age, was to improve maternal and child nutrition. The child ration (3 kg of CSB and 300 g of oil per month) provided 458 kcal/d. *Tubaramure*'s second component focused on health and sought to improve the provision of preventive health services and to increase the use of these services by pregnant and lactating women and by children 0 to 23.9 months of age. The program's third component, the BCC strategy, was designed to promote adequate health, hygiene, and nutrition behaviors and practices. The BCC was implemented by program staff, locally hired *Tubaramure* health promoters, and leader mothers who were selected as teachers by their fellow beneficiary mothers. *Tubaramure* beneficiary mothers were expected to participate in the BCC sessions and to attend preventive health services, but neither expectation was enforced as a condition for receiving the food rations. Further details on the program and its impacts on a range of outcomes have been described elsewhere (11–14), and are also provided in the **Supplemental Text**.

### Evaluation design

The program evaluation used a cluster-randomized, controlled design whereby 60 collines (a colline or hill is an official administrative unit in Burundi) meeting the study criteria were randomly assigned to 1 of 4 study arms. Collines were eligible if they fell between the first and 99th percentiles for colline population size and were not served by >1 health center each. A cluster design was used since individual randomization of the *Tubaramure* program components was not feasible. Prior to randomization, 210 collines meeting the study criteria were grouped into strata based on population size. The number of strata in each province (5 in Cankuzo, 10 in Ruyigi) reflected the relative population size. Each stratum had 13 or 14 collines in Cankuzo and 14 or 15 collines in Ruyigi. Using random numbers with a fixed random number seed (Stata version 11, StataCorp 2009) (20), 4 collines were randomly drawn from each stratum, for a total of 60 collines. At a public lottery event with representatives from both provinces, the 4 collines in each stratum were randomly assigned to the 4 different study arms: 3 treatment arms and 1 control arm. The study included 3 treatment arms to assess the differential effects of varying the timing (starting during pregnancy or at birth) and duration (full 1000 days or shorter) of receiving food rations. The rationale for studying these was that providing food rations for the full 1000 days period is expensive; if a similar impact is obtained when providing rations for a shorter time period, more beneficiaries can be covered with the same resources.

Beneficiaries in the T24 arm received the standard program: that is, all program benefits during pregnancy and up to the age of 23.9 months for the child. Beneficiaries in the T18 arm received the same benefits, but food rations ended at the age of 17.9 months for the child. In the TNFP arm (no food during pregnancy), food rations started only at birth and were provided to the mother for the first 6 months and to the child between 6 and 23.9 months of age. Collines assigned to the control arm did not receive any *Tubaramure* benefits, but had access to the standard health-care services provided by the Ministry of Health. Since health centers were used by various collines, the health service intervention component was not limited to the treatment arms.

We conducted 3 repeated cross-sectional surveys: a baseline survey in 2010 (before the program started) and 2 follow-up surveys in 2012 and 2014 (**Table 1**). Each follow-up survey was conducted to assess a specific set of outcomes. Outcomes such as anemia and wasting were best measured when children were still eligible to receive program benefits: that is, when they were between 0 to 23.9 months of age. Thus, these outcomes were assessed using data from the baseline surveys (conducted before the program started) and the 2012 follow-up survey (when children 0 to 23.9 months of age were eligible to participate in the ongoing program). The full effect on child linear growth—the main outcome of the study—was expected in children who had been exposed to *Tubaramure* from early pregnancy to when they reached 23.9 months of age. Impacts on child linear growth were therefore assessed among children 24 months and older, using data from the baseline survey and the 2014 follow-up survey (when the program had ended and children were between 24 and 41.9 months of age) (14). The first follow-up thus included households with children 0 to 23.9 months of age, the second follow-up included households with children 24 to 41.9 months of age, and the baseline survey included both types of households.

**TABLE 1:** Survey waves and *Tubaramure* program implementation

	Survey rounds		
	Baseline, 2010	Follow-up, 2012	Follow-up, 2014
Inclusion in survey: households with …			
Child 0 to 23.9 mo of age	Yes	Yes	No
Child 24 to 41.9 mo of age	Yes	No	Yes
*Tubaramure* program being implemented?	No, started after the baseline survey	Yes	No, program ended before the 2014 follow-up
Child nutrition outcomes of primary focus in …			
Children 0 to 23.9 mo of age	Weight-for-height, wasting, anemia	Weight-for-height, wasting, anemia	—
Children 24 to 41.9 mo of age	Linear growth, stunting	—	Linear growth, stunting

The 2010 and 2012 data on children 0 to 23.9 months of age were used in the analyses presented in this manuscript. Shortly after the baseline survey was completed, eligible families were invited to enroll in *Tubaramure*, and beneficiaries started receiving program benefits.

The study in children 0 to 23.9 months of age was powered to detect a program effect (difference between treatment and control) on child anemia (1 of the study's primary outcomes) in children of 11 percentage points (pp) in the T24 and TNFP groups and of 8.25 pp in the T18 group using a Type 1 error (α) of 0.05 (1-sided), a power of 0.90, an intracluster correlation coefficient of 0.006, and 15 clusters per treatment arm (13). The differences in expected effect size thus resulted in different sample sizes across arms. Using the same parameters and a baseline prevalence of wasting of 7%, we calculated that the study's sample size allowed us to detect a reduction in the prevalence of wasting of 4 pp and a change in the mean of weight-for-length *z*-score (WLZ) of 0.24 when comparing the T24 or TNFP arm to the control group. For the T18 to control comparison, the detectable differences for wasting and WLZ were 4 pp and 0.21, respectively. Since all statistical models controlled for covariates, the actual minimum detectable differences were smaller.

The International Food Policy Research Institute's Institutional Review Board and the Ministry of Health of Burundi approved the study. Written informed consent for participation in the study was obtained before the start of each interview. This trial was registered at clinicaltrials.gov as NCT01072279.

### Data sources and measurement

**FIGURE 1:**
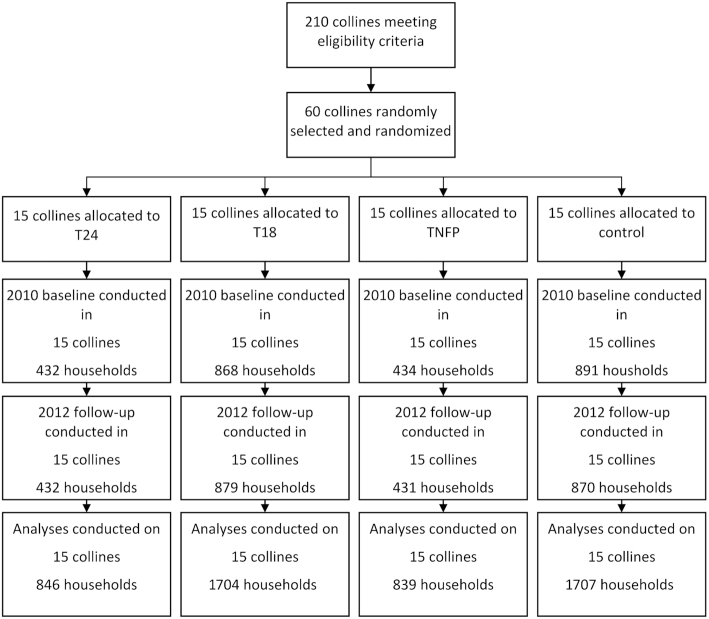
Trial flow chart. Abbreviations: T18, treatment arm from pregnancy to 18 months; T24, treatment arm from pregnancy to 24 months; TNFP, treatment arm from birth to 24 months.

At the start of the 2010 and 2012 surveys, a household census was conducted in all 60 research collines to generate a complete list of households with a child aged 0 to 23.9 months. Using a probability proportional to size approach, we calculated the target sample size for each colline. Colline-specific lists of randomly ordered households to be surveyed were then generated. Households were visited in the order listed until the required sample size in each colline was reached. As our objective was to estimate the intent-to-treat effect, inclusion in the survey was solely based on having a child in the appropriate age group and not on actual program participation. If there was more than 1 child in this age group, 1 “index child” was randomly chosen using the alphabetic order of the children's first names. A total of 2625 and 2612 households with a child aged 0 to 23.9 months were surveyed at baseline and follow-up, respectively (**Figure 1**).

The survey team was trained extensively, which included classroom teaching, field exercises, and repeated testing to assess skill acquisition. Each field team was composed of 4 enumerators, 2 anthropometrists, and 1 team controller. The enumerators used a household questionnaire to collect data on a wide range of variables, including socio-demographic characteristics (such as education, literacy, and household asset ownership) and program participation. Nurses were trained [and standardized (21)] to collect anthropometric data. Length and height were collected using Shorr boards (Weight and Measure). Measurements were taken twice, and were taken a third time if the difference between the first 2 measurements exceeded 6 mm. The 2 closest measurements were used in the analyses. Weight data were collected using a Seca 874 digital scale (Seca), which allowed the weight of the child to be taken when the mother held the child. WLZ was calculated using the WHO 2006 growth standard (22). Wasting was defined as WLZ <2SD.

### Statistical analyses

In line with the CONSORT 2010 guidelines, no formal comparison of baseline means between the treatment and intervention arm was conducted (23). The impact of *Tubaramure* was estimated using a double-difference colline–fixed effect model, which estimates changes over time in the treatment group relative to the control group. This model was used:

(1) $$\begin{equation*}
{y_{t = 0,1}} = {\beta _0} + {\beta _1}{T_j} + {\beta _2}{S_i} + {\beta _3}{T_j}{S_i} + {\beta _4}C + {\beta _i}{X_i} + \varepsilon
\end{equation*}$$

Here, *T_j_* is time (baseline or follow-up), *S_i_* is the assigned study arm (T24, T18, TNFP, or control), and *C* is a vector representing the colline*-*level fixed effects. The coefficient β_3_ represents the estimated treatment effect. Colline-level fixed effects were used to control for unobserved time-invariant colline characteristics at baseline and follow-up. To reduce residual noise and thus maximize power, covariates (*X_i_*) were added to the model. These included maternal and child age, child sex, maternal height, whether the primary caregiver was the biological mother (and the interaction between this variable and maternal height), the education levels of the mother and the head of household, dependency ratio, and household assets. In line with statistical theory, we used 1-sided tests given the a priori hypothesis that the program would lead to improvements in nutritional status (24).

In previous analyses, we found that the impact of the program on linear growth faltering was limited to children growing up in wealthier households, to children with literate mothers, and to children with better-schooled parents. These differences were not due to differences in program enrollment or participation in program activities (14). To assess whether the impact on wasting varied by socio-economic characteristics, we estimated the impact models separately by level of maternal education (none vs. some), maternal literacy (illiterate vs. literate), education of the head of household (none vs. some), and household asset ownership (below or above median number of assets, a proxy for socio-economic status).

A similar approach was used to evaluate whether the impact differed by child age: separate models were estimated for children 0 to 5.9 months, 6 to 11.9 months, 12 to 17.9 months, and 18 to 23.9 months of age. All treatment arms were pooled in the subgroup analyses.

The SEs of all estimated parameters were adjusted for colline-level clustering by using a clustered (Huber-White) sandwich estimator. A *P* value of 0.05 was considered significant. Analyses were conducted using Stata 16 (StataCorp, version 16) (20). All analyses pertain to the individual level. No clusters were dropped from the analyses. Fewer than 3% of individual observations were excluded from the analysis because of missing or incomplete data (Figure 1).

## Results

Education levels in the sample were very low (**Table 2**). Fewer than 10% of household heads had completed primary school. Similarly low schooling levels were found among the mothers of the study children, with less than 6% having completed primary school. At the 2012 follow-up survey, the majority of households in the treatment arms reported being either a current or past *Tubaramure* beneficiary. Nearly all beneficiary households participated in the monthly food distributions: the average number of distributions attended in the past 4 months was close to (the expected) 4 (14). Participation in care group sessions was much lower: mothers reported having attended fewer than 4 care groups in the past 4 months (as compared to the expected 8). Home visits to beneficiary households by the leader mother were rare. Baseline characteristics were well balanced across trial arms (Table 2).

**TABLE 2:** Descriptive statistics for households with a child 0 to 23.9 months of age at baseline (2010) and follow-up (2012) participating in the *Tubaramure* evaluation in Cankuzo and Ruyigi (Burundi)

	Baseline, 2010						Follow-up, 2012					
		Study arm						Study arm				
	Full sample	All T arms	T24	T18	TNFP	Control	Full sample	All T arms	T24	T18	TNFP	Control
*n*	2530	1678	421	838	419	852	2566	1711	425	866	420	855
Household members	5.6 ± 2.1	5.6 ± 2.0	5.6 ± 2.0	5.7 ± 2.0	5.7 ± 2.1	5.5 ± 2.1	5.8 ± 2.0	5.8 ± 2.0	5.8 ± 1.9	5.7 ± 2.0	5.8 ± 2.1	5.8 ± 2.0
Head of household education												
None/preschool, %	40.3	39.4	39.0	40.2	38.2	42.0	37.8	35.5	35.1	34.4	38.3	42.5
Primary incomplete, %	53.9	54.1	55.3	53.3	54.2	53.6	55.9	57.2	58.6	58.2	53.8	53.3
Primary complete, %	5.8	6.6	5.7	6.4	7.6	4.3	6.2	7.2	6.4	7.4	7.9	4.2
Head of household occupation												
Farms own or family land, %	76.2	75.3	77.0	75.3	73.5	78.2	76.1	74.6	75.3	74.4	74.3	79.1
Farms someone else's land/aglabor/unemployed, %	10.2	8.9	7.6	9.4	9.3	12.6	9.2	7.9	7.1	8.9	6.7	11.7
Manual labor, %	5.5	7.0	6.9	7.4	6.2	2.7	5.9	6.8	8.0	6.7	6.0	4.1
Other	8.1	8.8	8.6	7.9	11.0	6.6	8.8	10.7	9.6	10.0	13.1	5.1
Mother												
Age, y	28.6 ± 7.0	28.8 ± 7.0	28.3 ± 6.9	29.0 ± 7.2	28.7 ± 6.7	28.3 ± 7.0	28.5 ± 6.6	28.7 ± 6.5	28.6 ± 6.3	28.5 ± 6.5	29.1 ± 6.9	28.2 ± 6.6
Literate, %	51.9	52.9	55.8	52.6	50.6	49.8	55.9	59.8	63.1	60.0	56.0	48.2
Maternal education												
None/preschool, %	51.9	50.8	50.8	49.2	54.2	54.0	46.3	43.0	42.8	40.9	47.6	52.9
Primary incomplete, %	44.4	44.7	44.7	47.1	39.9	43.8	49.5	52.1	52.5	54.0	47.6	44.4
Primary complete, %	3.7	4.5	4.5	3.7	6.0	2.2	4.2	4.9	4.7	5.1	4.8	2.7
Child												
Age, mo	12.8 ± 6.8	12.6 ± 6.8	12.3 ± 7.1	12.9 ± 6.7	12.4 ± 6.6	13.1 ± 6.8	12.3 ± 6.6	12.0 ± 6.6	12.1 ± 6.4	12.0 ± 6.8	11.9 ± 6.5	13.0 ± 6.5
Male, %	47.4	47.4	46.6	47.5	48.0	47.5	51.2	51.5	50.1	52.4	51.0	50.6
Wasted, %	7.0	7.3	5.7	7.7	7.9	6.5	6.0	5.1	5.6	4.2	6.4	8.0
Weight-for-length *Z*-score	−0.3 ± 1.2	−0.3 ± 1.2	−0.3 ± 1.2	−0.3 ± 1.2	−0.3 ± 1.2	−0.3 ± 1.2	−0.2 ± 1.2	−0.2 ± 1.1	−0.2 ± 1.1	−0.1 ± 1.2	−0.3 ± 1.1	−0.3 ± 1.2
Current *Tubaramure* beneficiary, %	—	—	—	—	—	—	45.2	65.9	73.8	55.2	79.8	2.4
Past *Tubaramure* beneficiary, %	—	—	—	—	—	—	9.4	13.2	6.4	20.5	5.1	1.7

Data are mean ± SD unless otherwise specified. The T24 arm received all program benefits during pregnancy and up to the age of 23.9 months for the child. The same benefits were received in the T18 arm, but food rations were discontinued when the child was 17.9 months of age. The TNFP (no food during pregnancy) arm started receiving food rations at birth, but the other benefits were the same as in T24. Abbreviations: T, treatment; T18, treatment arm from pregnancy to 18 months; T24, treatment arm from pregnancy to 24 months; TNFP, treatment arm from birth to 24 months.

**FIGURE 2:**
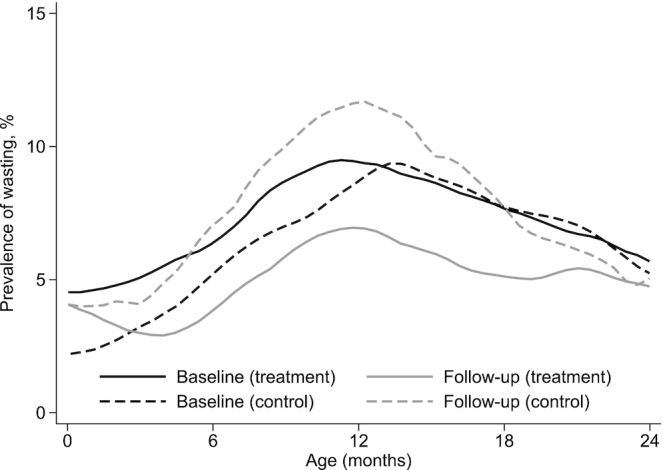
Prevalence of wasting at baseline (2010) and at follow-up (2012) by child age in children 0 to 23.9 months participating in the *Tubaramure* evaluation in Cankuzo and Ruyigi (Burundi). Kernel-weighted local polynomial smoothing was used to calculate the age-specific prevalence (baseline treatment, *n* = 1668; baseline control, *n* = 847; follow-up treatment, *n* = 1711; follow-up control, *n* = 855).

The prevalence of wasting at baseline was 7% [considered a medium prevalence (25)], and the mean WLZ was −0.3. In both the treatment and control arms, prevalence of wasting was lowest at birth and reached a peak prevalence of around 9% at the age of 12 months, after which it declined again (**Figure 2**). A similar age pattern was found at follow-up, but the prevalence of wasting decreased in all 3 treatment arms between baseline and endline (to 5.1% for treatment arms combined), whereas it increased in the control arm (to 8%). Prevalence at 12 months in the control arm was well above 10% [considered high (25)].

**FIGURE 3:**
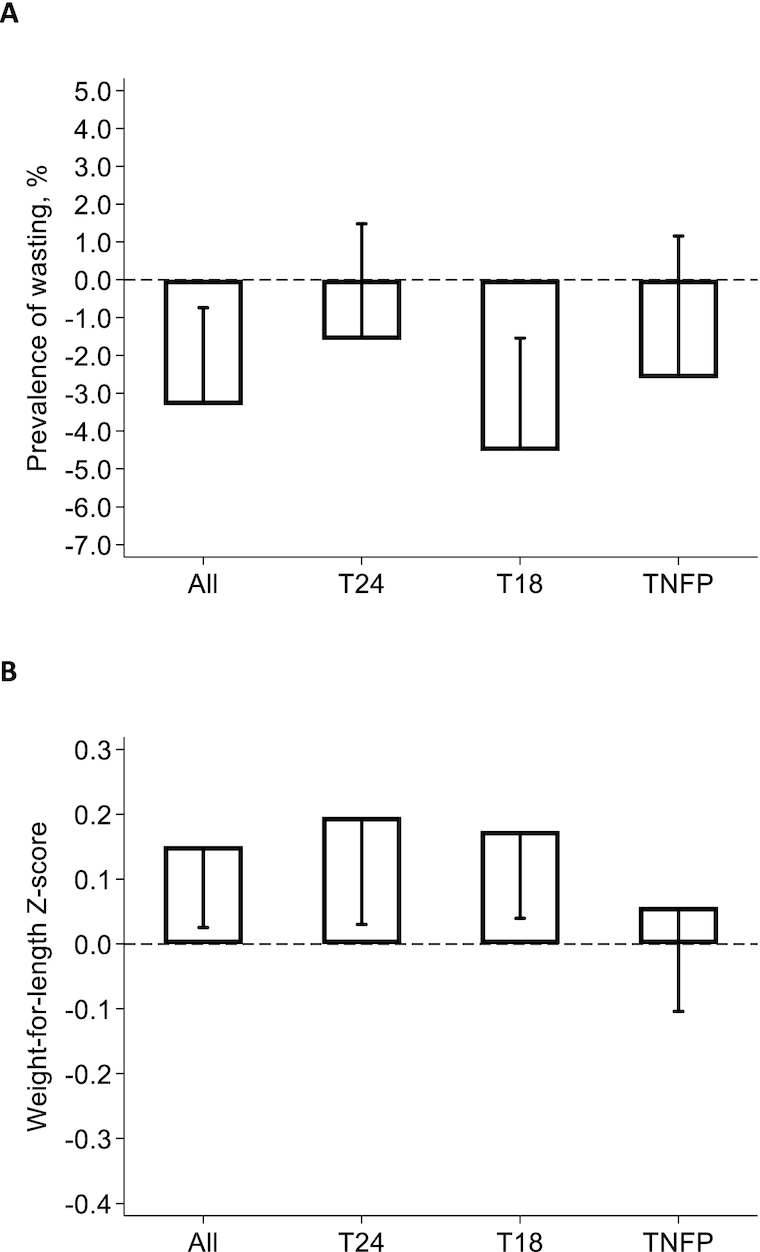
Impact of *Tubaramure* on wasting (A) and weight-for-length *z*-score (B) in children 0 to 23.9 months in Cankuzo and Ruyigi (Burundi). Difference-in-difference impact and 1-sided 95% confidence bound (adjusted for clustering at the locality level using the Huber-White sandwich estimator) were estimated using cluster fixed effects models. Covariates in the difference-in-difference model included maternal and child age, child sex, maternal height, whether the primary caregiver was the biological mother (and the interaction between this variable and height), education levels of the mother and the head of household, dependency ratio, and household assets (*n* *=* 5081). The T24 arm received all program benefits during pregnancy and up to the age of 23.9 months for the child. The same benefits were received in the T18 arm, but food rations were discontinued when the child was 17.9 months of age. The TNFP (no food during pregnancy) arm started receiving food rations at birth, but the other benefits were the same as in T24. Complete regression results are shown in Supplemental Table 3. Abbreviations: T18, treatment arm from pregnancy to 18 months; T24, treatment arm from pregnancy to 24 months; TNFP, treatment arm from birth to 24 months.

*Tubaramure* had a statistically significant protective effect on the prevalence of wasting in the T18 arm (−4.5 pp, or a 56% reduction relative to control at endline) and when all intervention groups were combined (−3.3 pp, or a 41% reduction; **Figure 3**; **Supplemental Table 3**). No significant effect on wasting was found in either the T24 or TNFP arm. Significant effects on WLZ were found in the T24 (0.20) and T18 (0.17) arms. WLZ was found to increase by 0.15 as a result of the program when all arms were combined. There was no impact on WLZ in the TNFP arm.

**FIGURE 4:**
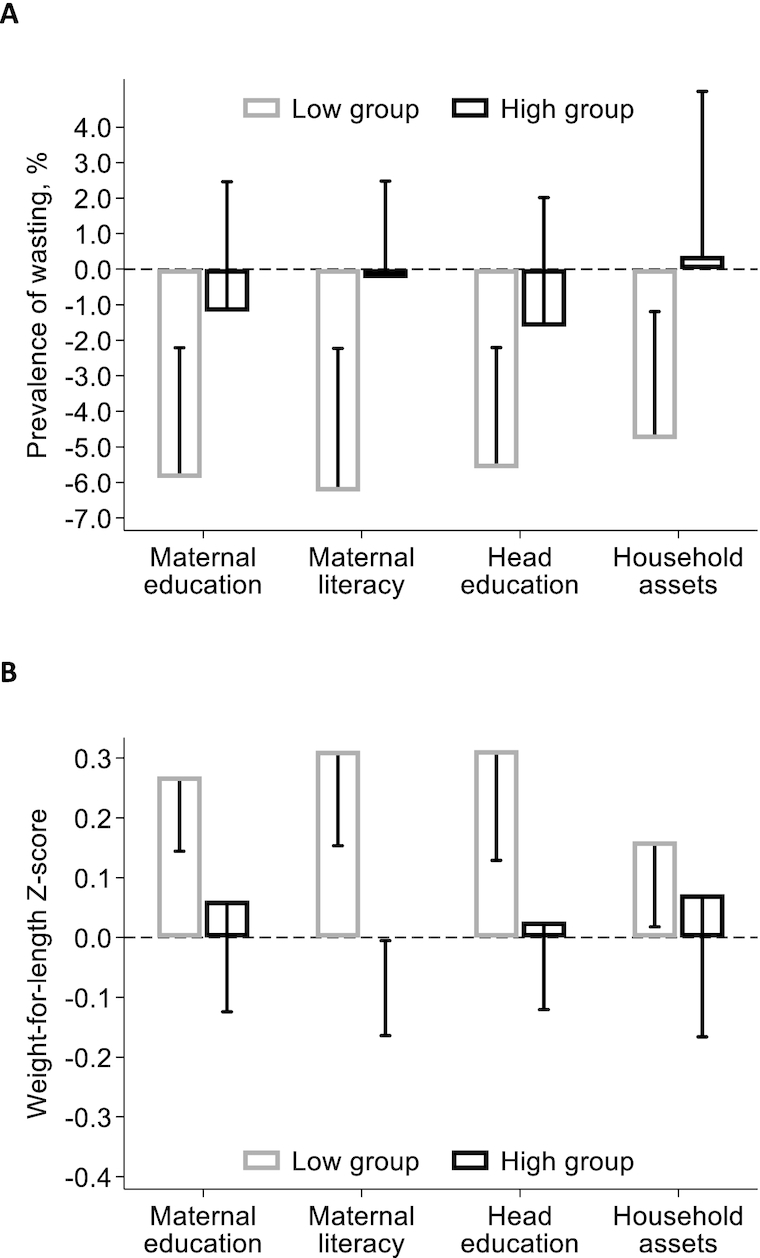
Impact of *Tubaramure* on wasting (A) and weight-for-length *z*-score (B) by maternal education and literacy, education of the head, and household assets in children 0 to 23.9 months in Cankuzo and Ruyigi (Burundi). Difference-in-difference impact and 1-sided 95% confidence bound (adjusted for clustering at the locality level using the Huber-White sandwich estimator) were estimated using cluster fixed effects models. Covariates in the difference-in-difference model included maternal and child age, child sex, maternal height, whether the primary caregiver was the biological mother (and the interaction between this variable and height), education levels of the mother and the head of household, dependency ratio, and household assets. Results are presented by maternal education level (no education vs. some; *n =* 2492 and 2589, respectively) and literacy (illiterate vs. literate; *n* = 2341 and 2740), by education level of the head of household (no education vs. some; *n* = 1983 and 3098), and by household assets (below vs. above median asset holdings; *n =* 2821 and 2260, respectively). The T24 arm received all program benefits during pregnancy and up to the age of 23.9 months for the child. The same benefits were received in the T18 arm, but food rations were discontinued when the child was 17.9 months of age. The TNFP (no food during pregnancy) arm started receiving food rations at birth, but the other benefits were the same as in T24. Full regression results are shown in Supplemental Table 5. Abbreviations: T18, treatment arm from pregnancy to 18 months; T24, treatment arm from pregnancy to 24 months; TNFP, treatment arm from birth to 24 months.

**FIGURE 5:**
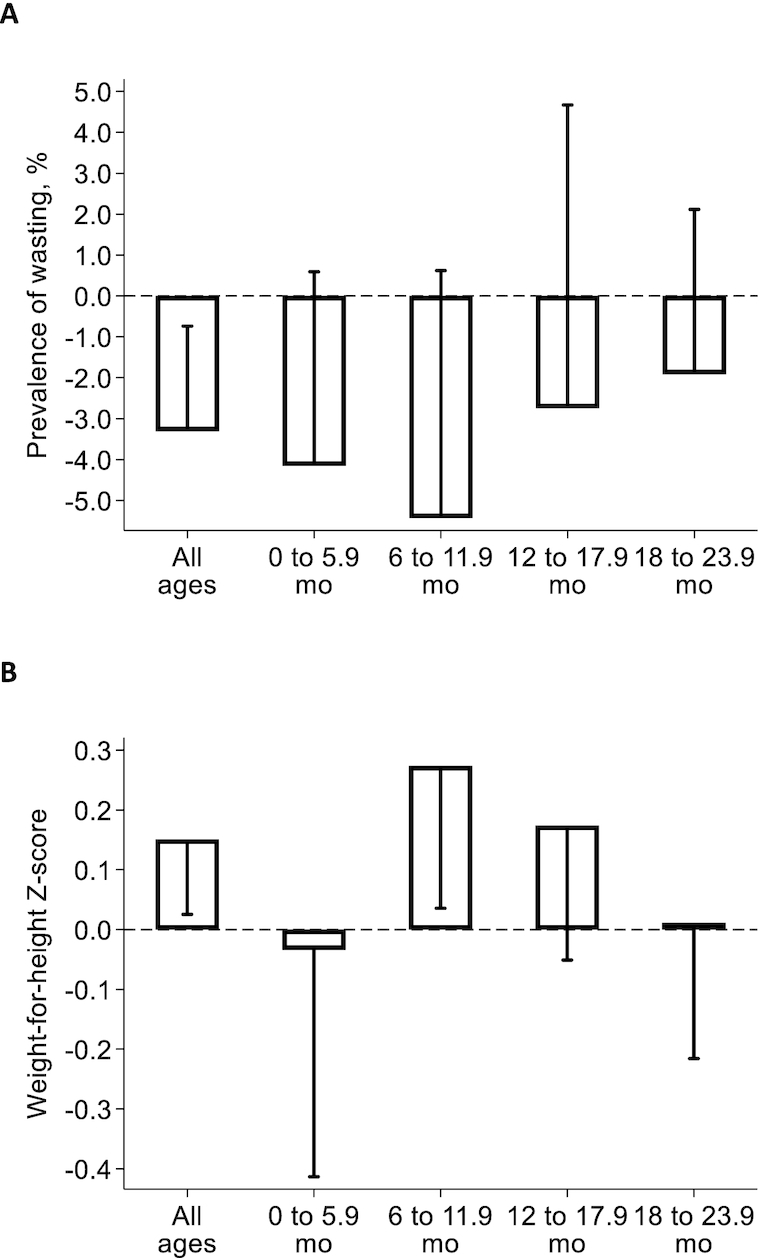
Impact of *Tubaramure* on wasting (A) and weight-for-length *z*-score (B) by child age in children 0 to 23.9 months in Cankuzo and Ruyigi (Burundi). Difference-in-difference impact and 1-sided 95% confidence bound (adjusted for clustering at the locality level using the Huber-White sandwich estimator) were estimated using cluster fixed effects models. Covariates in the difference-in-difference model included maternal and child age, child sex, maternal height, whether the primary caregiver was the biological mother (and the interaction between this variable and height), education levels of the mother and the head of household, dependency ratio, and household assets. Results are presented by child age (0 to 5.9, 6 to 11.9, 12 to 17.9, and 18 to 23.9 months; *n* = 1092, *n* = 1293, *n* = 1307, and *n* = 1389, respectively). The T24 arm received all program benefits during pregnancy and up to the age of 23.9 months for the child. The same benefits were received in the T18 arm, but food rations were discontinued when the child was 17.9 months of age. The TNFP (no food during pregnancy) arm started receiving food rations at birth, but the other benefits were the same as in T24. Full regression results are shown in Supplemental Table 5. Abbreviations: T18, treatment arm from pregnancy to 18 months; T24, treatment arm from pregnancy to 24 months; TNFP, treatment arm from birth to 24 months.

At baseline, the prevalence of wasting was higher in households where mothers had lower, compared to higher, levels of education and in households where the mother was illiterate (compared to literate). No differences in prevalence were found by education level of the head of household or by the level of household asset holdings (**Supplemental Figure 1;Supplemental Table 4**). At follow-up, the prevalence of wasting had increased in the control arm and clear differences by socio-economic characteristics had emerged: the prevalence of wasting in children growing up in the most disadvantaged conditions was considerably higher than that in children in better-off households. Our subgroup analysis (**Figure 4**; **Supplemental Tables 5** and **6**) shows that the impact on wasting was statistically significant only in the most disadvantaged children. The impact analyses by child age indicate that the largest impact was found in children 6 to 12 months of age: that is, around the time when the prevalence of wasting peaked in this population (**Figure 5**; Supplemental Tables 5 and 6).

## Discussion

Using a cluster-randomized, controlled trial, we demonstrated that *Tubaramure*, a FA-MCHN program in Burundi, reduced child wasting by approximately half. The absence of a significant effect in the study group that did not receive the program during pregnancy (TNFP arm) suggests that exposure to the food rations during pregnancy contributed to the program's impact on child wasting. The cross-sectional design, unfortunately, does not allow us to study this hypothesis in more depth.

The magnitude of *Tubaramure*’s impact was large. A recent meta-analysis of 7 randomized controlled food supplementation and feeding trials in young children found a nonsignificant mean effect on WLZ of 0.10 SD (26). The prevalence of wasting was not assessed in this review. A variety of supplements (including milk-based formula, milk- and soy-based fortified spreads, and fortified complementary foods) providing 285 to 750 kcal per day were used in the included studies, and treatment duration varied from 3 to 24 months. A more recent meta-analysis with stricter study inclusion criteria found that complementary food supplementation interventions with or without nutrition education had a small yet statistically significant effect in food-insecure settings on WLZ of 0.05 SD (3). Different supplements provided 118 to 285 kcal per day for 9 to 12 months. The studies included in both reviews typically provided close supervision of adherence. Since our study was designed as a program effectiveness trial, no supervision was provided. The larger effect size in our trial (as compared to the findings from the meta-analyses) could be related, at least in part, to the larger ration targeted to children (458 kcal/d) and the supplementation during pregnancy in Burundi.

Our second study objective was to assess whether the impact of the program varied by socio-economic characteristics and child age. The effects on wasting and WLZ were limited to children who had illiterate mothers or mothers who had received no education and to children in the worst-off households, defined as those that had fewer assets or had a head without any education. In these children, the program lowered wasting by 5 to 6 pp and increased WLZ by 0.2 to 0.3. The worst-off children who did not receive the intervention (control arm) saw an increase in the prevalence of wasting of 3 to 5 pp between baseline to follow-up (Supplemental Table 5). This was most likely a consequence of the severe deterioration of the food security and nutrition situation in the study area (11, 19). The program thus effectively protected the most disadvantaged children from the worsening conditions. Our analyses by age suggest that the largest effect was found in children 6 to 12 months of age. This is the age leading up to the peak incidence of wasting in this population, thus strengthening the plausibility of our findings.

Interestingly, we previously documented that the program's effect on stunting was concentrated in children who were relatively better off (14): that is, the group in which no effect on wasting was found. We found no evidence of differences in program enrollment or in participation in program activities between these socio-economic groups (14). Morbidity symptoms, fever, and diarrhea (as reported by the caregiver) were not different across these groups either (**Supplemental Figures 2**–**4**). We hypothesized in our stunting impact manuscript that the program “dosage” may have been too low for the most disadvantaged children to biologically respond in terms of linear growth (14). Inadequate protein and micronutrient intake may have limited these children's ability to gain length while still allowing them to gain weight, thus reflecting fundamentally different biological mechanisms underlying the processes. Other factors, such as repeated infections (which caregivers may not have been able to observe) or environmental enteric dysfunction, could have prevented these children from growing in length but did not stop them from benefitting in terms of weight (27). We cannot explore these underlying mechanisms, as no detailed dietary intake or biomarker data were collected in this study.

At the time the study was conducted, there was an alarming increase in inflation of consumer prices (19) and, correspondingly, a steep increase in food insecurity in the study area (11). In the same period, worrisome increasing trends were observed in the prevalences of maternal and child anemia (13) and child stunting (14). The general worsening of the nutrition situation in the study area between 2010 and 2012 was also observed in the prevalence of child wasting: in children 12 months of age (the age of peak incidence in this population), the prevalence increased from around 9 to nearly 12% in the control group, while it dropped from 9 to 7% in the treatment arms (Figure 2). Our findings show that the *Tubaramure* program successfully protected children from the deteriorating conditions in Burundi. This is in line with our previous findings, which documented the program's significant impact on maternal and child hemoglobin and anemia (13), stunting (14), household consumption, and maternal and child diets (11). A similar FA-MCHN program in Haiti was also previously shown to mitigate the negative effects of an ongoing economic crisis (28). Together, these findings show that FA-MCHN programs provide an effective strategy to protect vulnerable households, women, and children from the negative effects of economic shocks. These results are particularly relevant in the context of the COVID-19 pandemic and the global economic crisis it has caused. A recent analysis of Demographic Health Survey data on 1.256 million children from 52 countries showed that economic shocks were associated with large increases in the prevalence of wasting. A 10% decline in the gross per capita national income was estimated to lead to increases in moderate/severe (WHZ <2 SD) and severe (WHZ <3 SD) wasting of 14 and 22%, respectively (15). It is thus expected that the pandemic-related income shocks will lead to a sharp increase in the prevalence of acute malnutrition in young children.

Evidence on what works to prevent wasting is limited, as research on wasting has focused more on the development of efficacious and effective treatment strategies in emergencies than on identifying interventions to prevent this form of malnutrition (1, 3). Given the high risk of mortality and the complexity and high cost of treatment, it is imperative to find effective interventions to prevent wasting (29, 30). Our study is among the first to document that FA-MCHN programs can help mitigate the negative effects of economic downturns on childhood wasting and protect the most vulnerable children.

The cross-sectional study design limits our ability to identify and quantify the pathways of impact. Our previous analyses, however, provide plausible evidence that the effect operated through improvements in child diet and reductions in morbidity. *Tubaramure* increased the likelihood of children 6 to 24 months of age achieving the minimum recommended dietary diversity and meal frequency (11). The program also improved the percentage of food-secure households, and increased household energy and micronutrient consumption. The effects on many of these outcomes were attributable to the food rations. *Tubaramure* also lowered the prevalence of symptoms of illness and fever, as reported by the mother (13). Since the study was designed to evaluate the combined impact of food aid, health, and BCC components, we cannot quantify the relative importance of these components individually.

In conclusion, this study shows that FA-MCHN programs are an effective tool to protect the most vulnerable children from wasting following shocks. Our findings are particularly important in the context of the economic crisis triggered by the COVID-19 pandemic, which has been predicted to lead to a steep increase in wasting prevalence if no programmatic response is put in place.

## Supplementary Material

nxaa330_Supplemental_File

## Notes

The study was made possible by the generous support of the American people through the support of the Office of Food for Peace, Bureau for Democracy, Conflict, and Humanitarian Assistance, and the Office of Health, Infectious Diseases, and Nutrition, Bureau for Global Health, U.S. Agency for International Development (USAID), under terms of Cooperative Agreement No. AID-OAA-A-12-00005, through the Food and Nutrition Technical Assistance III Project (FANTA), managed by FHI 360. This study also received support from the CGIAR Research Program on Agriculture for Nutrition and Health (A4NH), led by the International Food Policy Research Institute. The research took place within the US Agency for International Development's Food for Peace–funded *Tubaramure* program. This program was implemented by Catholic Relief Services in consortium with Food for the Hungry, International Medical Corps, and Caritas Burundi.

Author disclosures: JLL is a member of the *Journal of Nutrition*’s Editorial Board. All the other authors report no conflicts of interest.

Supplemental Text, Supplemental Tables 1–6, and Supplemental Figures 1–4 are available from the “Supplementary data” link in the online posting of the article and from the same link in the online table of contents at https://academic.oup.com/advances/.

Abbreviations used: BCC, behavior change communication; COVID-19, coronavirus disease 2019; CSB, corn-soy blend; FA-MCHN, food-assisted maternal and child health and nutrition; pp, percentage points; T18, treatment arm from pregnancy to 18 months; T24, treatment arm from pregnancy to 24 months; TNFP, treatment arm from birth to 24 months; WLZ, weight-for-length *z*-score.
