# Supplementary material for: Tubaramure, a Food-Assisted Integrated Health and Nutrition Program, Reduces Child Wasting in Burundi: A Cluster-Randomized Controlled Intervention Trial
Source: J Nutr. 2020 Nov 26;151(1):197–205. doi: 10.1093/jn/nxaa330 (PMC7717329; doi:10.1093/jn/nxaa330)
Supplement: nxaa330_Supplemental_File [file nxaa330_supplemental_file.docx]

**Supplemental Text**^[[1]](#footnote-1)^

**The three program components of the Tubaramure program**

The core package of the *Tubaramure* program included three components: the distribution of food rations; improvements in the provision and use of health services; and a behavior change communication (BCC) strategy focused on improving health, hygiene and nutrition practices. The **food** component of the *Tubaramure* program aimed to increase household food security in terms of both quantity and quality (through a family ration containing micronutrient-fortified foods), and maternal and child nutrition (through the individual micronutrient-fortified foods ration targeted at pregnant and lactating mothers and children aged from 6 mo (up to 17.9 or 23.9 mo depending on the study group)). The monthly household food ration provided to all *Tubaramure* beneficiary households included 12 kg of corn–soy blend (CSB) and 1,200 g of vitamin A- and D-fortified vegetable oil (see **Supplemental Table 1** for micronutrient composition). An individual ration was provided in addition to the household food ration. For beneficiary mothers during pregnancy and during the first 6 mo of lactation, this ration included 6 kg of CSB and 600 g of oil; the child’s individual ration, which was provided starting at 6 mo – the age at which food should be introduced in the child’s diet in addition to breast milk – included 3 kg of CSB and 300 g of oil. Decisions about the types and quantities of food included in the rations were made by the consortium of NGOs.

The **health** component was designed to improve the provision of preventive health services by health staff and to increase the use of these services by pregnant and lactating women and children aged between 0 and 23.9 mo. The *Tubaramure* program trained health staff and provided some key supplies for implementing health services (e.g., equipment for prenatal care, labor and delivery, growth monitoring, and curative care). In addition, the program encouraged the use of preventive health services by pregnant and lactating women (i.e., pre and postnatal services, respectively) and children aged 0–23.9 mo (i.e., growth monitoring and promotion, vaccination) through the BCC strategy. Beneficiary mothers and children were expected to attend and use these recommended preventive health services at the local health center but attendance was not set as a condition for receipt of the program’s food assistance.

*Tubaramure*’s third component, the **BCC strategy**, was designed to encourage the adoption of best practices in health, hygiene, and nutrition. It was implemented by program staff, locally hired *Tubaramure* health promoters (THPs), and leader mothers who were selected as teachers by their fellow beneficiary mothers. The curriculum contained five modules, each with between 6 and 12 lessons. Lessons provided mothers with information on essential nutrition, hygiene and care practices during pregnancy (e.g., maternal nutrition and handwashing) and during infancy and young childhood (e.g., infant and young child feeding practices, danger signs of childhood illness and point-of-use water treatment), on the management of childhood illness (e.g., use of ORS and feeding of sick children), and on the importance of using bed nets. The *care groups* for leader mothers were held every 2 wk. During these group meetings, leader mothers were trained by the THP. Leader mothers in turn met with the beneficiary mothers in beneficiary mother care groups every 2 wk and discussed the topics they had most recently learned from the THP. Both types of care groups were supposed to consist of 10 to 12 mothers. As with the health services, beneficiary mothers were expected to attend the BCC sessions, but this was not enforced as a condition of receiving the food ration.

**Supplemental Figure 1:** Prevalence of wasting at (A) baseline (2010, all study arms) and (B) at follow-up (2012, control arm) by socio-economic characteristics in children 0 to 23.9 mo in Burundi

**A**

**B**

**Supplemental Figure 2:** Prevalence of any morbidity symptoms in the past two weeks (as reported by the caregiver) at (A) baseline (2010, all study arms) and (B) at follow-up (2012, control arm) by socio-economic characteristics in children 0 to 23.9 mo in Burundi

**A**

**B**

**Supplemental Figure 3:** Prevalence of fever in the past two weeks (as reported by the caregiver) at (A) baseline (2010, all study arms) and (B) at follow-up (2012, control arm) by socio-economic characteristics in children 0 to 23.9 mo in Burundi

**A**

**B**

**Supplemental Figure 4:** Prevalence of any type of diarrhea in the past two weeks (as reported by the caregiver) at (A) baseline (2010, all study arms) and (B) at follow-up (2012, control arm) by socio-economic characteristics in children 0 to 23.9 mo in Burundi

**A**

**B**

**Supplemental Table 1: Micronutrient concentrations of corn-soy blend (CSB) and fortified vegetable oil**

|  | **CSB** | **Vegetable oil** |
| --- | --- | --- |
| Iron, mg/100 g | 17.49 | 0.05 |
| Zinc, mg/100 g | 5.00 | 0.01 |
| Vitamin A, μg RE/100 g | 784 | 1,800–2,250 |
| Folic acid, μg/100 g | 300 | - |
| Vitamin C, mg/100 g | 40 | - |
| Cu, mg/100 g | 0.90 | - |
| Calcium, mg/100 g | 831 | - |
| Phosphorous, mg/100 g | 206 | - |
| Potassium, mg/100 g | 634 | - |
| Magnesium, mg/100 g | 173.8 | - |
| Selenium, mg/100 g | 6 | - |
| Iodine, μg/100 g | 56.9 | - |
| Manganese, mg/100 g | 0.7 | - |
| Sodium, mg/100 g | 7.3 | - |
| Thiamin, mg/100 g | 0.53 | - |
| Riboflavin, mg/100 g | 0.48 | - |
| Niacin, mg/100 g | 6.23 | - |
| Pantothenic acid, mg/100 g | 3.4 | - |
| Vitamin B-6, mg/100 g | 0.5 | - |
| Vitamin B-12, μg/100 g | 1 | - |
| Vitamin D, IU/100 g | 198 | 1,700–2,100 |
| Vitamin E, mg/100 g | 8.70 | 8.18 |
| Vitamin K, μg/100 g | - | 183.9 |

**Supplemental Table 2: Calculation of the per adult equivalent energy provided by the complete *Tubaramure* food ration**

| **Beneficiary type in household** | **CSB per month** | | **Oil per month** | | **Total food ration** | | |
| --- | --- | --- | --- | --- | --- | --- | --- |
|  |  |  |  |  | **Per month** | **Per day** | **Per adult equivalent per day**^1^ |
|  | **Quantity**  **(kg)** | **Energy**  **(kcal)** | **Quantity**  **(kg)** | **Energy**  **(kcal)** | **Energy**  **(kcal)** | **Energy**  **(kcal)** | **Energy**  **(kcal)** |
| Beneficiary type |  |  |  |  |  |  |  |
| Pregnant mother | 18 | 67,680 | 1.8 | 15,912 | 83,592 | 2,748 | 743 |
| Mother of child under 6 mo | 18 | 67,680 | 1.8 | 15,912 | 83,592 | 2,748 | 743 |
| Child 6 to 23.9 mo | 15 | 56,400 | 1.5 | 13,260 | 69,660 | 2,290 | 619 |
| “Average” Tubaramure household^2^ |  |  |  |  |  |  |  |
| T24 |  | 59,220 |  | 13,923 | 73,143 | 2,405 | 650 |
| T18 |  | 45,120 |  | 10,608 | 55,728 | 1,832 | 495 |
| TNFP |  | 59,220 |  | 13,923 | 73,143 | 2,405 | 650 |

^1^ The 2012 sample average of 3.7 adult equivalents per household was used.

^2^ Based on program participation data, we calculated a weighted sample average, using 6 months of participation during the first 6 mo of the child life and 18 mo from 6 to 23.9 mo in the T24 and TNFP arms and 12 months in T18.

**Supplemental Table 3**: Regression model to estimate the impact on wasting and WLZ in children 0 t0 23.9 mo in Burundi

|  | **Wasting** |  | **WLZ** |  |
| --- | --- | --- | --- | --- |
| **VARIABLES** | **Arms combined** | **By study arm** | **Arms combined** | **By study arm** |
|  | **All ages** | **All ages** | **All ages** | **All ages** |
|  |  |  |  |  |
| Wave | 1.20 | 1.20 | -0.03 | -0.03 |
|  | (1.32) | (1.32) | (0.07) | (0.07) |
| Wave x … |  |  |  |  |
| All T combined | -3.31** |  | 0.15** |  |
|  | (1.54) |  | (0.08) |  |
| T24 |  | -1.59 |  | 0.20** |
|  |  | (1.84) |  | (0.10) |
| T18 |  | -4.52*** |  | 0.17** |
|  |  | (1.79) |  | (0.08) |
| TNFP |  | -2.60 |  | 0.06 |
|  |  | (2.25) |  | (0.10) |
| Child |  |  |  |  |
| Age, mo | 1.10*** | 1.09*** | -0.17*** | -0.17*** |
|  | (0.24) | (0.24) | (0.01) | (0.01) |
| Age^2^, mo^2^ | -0.04*** | -0.04*** | 0.01*** | 0.01*** |
|  | (0.01) | (0.01) | (0.00) | (0.00) |
| Male | 3.13*** | 3.14*** | -0.12*** | -0.12*** |
|  | (0.74) | (0.74) | (0.04) | (0.04) |
| Mother |  |  |  |  |
| Age, y | 0.09 | 0.09 | -0.01*** | -0.01*** |
|  | (0.06) | (0.06) | (0.00) | (0.00) |
| Biological mother | -509.16 | -507.90 | 21.26** | 21.25** |
|  | (327.95) | (328.42) | (10.11) | (10.11) |
| Height, cm | -3.16 | -3.15 | 0.13** | 0.13** |
|  | (2.06) | (2.06) | (0.06) | (0.06) |
| Biological mother x height | 3.13 | 3.12 | -0.13** | -0.13** |
|  | (2.05) | (2.05) | (0.06) | (0.06) |
| Education |  |  |  |  |
| Primary incomplete | -1.11 | -1.12 | 0.06 | 0.06 |
|  | (0.75) | (0.75) | (0.04) | (0.04) |
| Primary complete | -2.51* | -2.48* | 0.19** | 0.19** |
|  | (1.49) | (1.48) | (0.09) | (0.09) |
| Head of household education |  |  |  |  |
| Primary incomplete | 0.55 | 0.56 | 0.01 | 0.01 |
|  | (0.87) | (0.87) | (0.03) | (0.03) |
| Primary complete | -1.85 | -1.86 | 0.20** | 0.20** |
|  | (1.19) | (1.20) | (0.08) | (0.08) |
| Household |  |  |  |  |
| Dependency ratio | -0.16 | -0.16 | -0.04 | -0.05 |
| Tertile 2 | (1.01) | (1.01) | (0.05) | (0.05) |
|  | 0.27 | 0.28 | 0.03 | 0.03 |
| Tertile 3 | (1.29) | (1.29) | (0.06) | (0.06) |
|  |  |  |  |  |
| Assets above median | -0.66 | -0.64 | 0.11*** | 0.11*** |
|  | (0.72) | (0.72) | (0.03) | (0.03) |
| Constant | 512.23 | 510.88 | -20.30* | -20.29* |
|  | (328.20) | (328.63) | (10.17) | (10.17) |
|  |  |  |  |  |
| Observations | 5,081 | 5,081 | 5,081 | 5,081 |
| R-squared | 0.02 | 0.02 | 0.09 | 0.09 |
| Number of collid | 60 | 60 | 60 | 60 |
|  |  | 0.111 |  | 0.799 |
|  |  | 0.660 |  | 0.177 |

Robust standard errors in parentheses; *** p<0.01, ** p<0.05, * p<0.1; one-sided tests for impact estimates.

**Supplemental Table 4**: Wasting and weight-for-length Z-score of children 0 to 23.9 mo of age at 2010 baseline (2010) and follow-up (2012)^1^

|  | **2010 baseline (2010)** | | | | | | **Follow up (2012)** | | | | | |
| --- | --- | --- | --- | --- | --- | --- | --- | --- | --- | --- | --- | --- |
|  | **Full sample** | **Study arm** | | | | | **Full sample** | **Study arm** | | | | |
|  |  | **All T arms** | **T24** | **T18** | **TNFP** | **Control** |  | **All T arms** | **T24** | **T18** | **TNFP** | **Control** |
| *n* | 3390 | 2446 | 943 | 553 | 950 | 944 | 3456 | 2907 | 963 | 968 | 976 | 549 |
| Child wasted, % |  |  |  |  |  |  |  |  |  |  |  |  |
| All | 7.0 | 7.3 | 5.7 | 7.7 | 7.9 | 6.5 | 6.0 | 5.1 | 5.6 | 4.2 | 6.4 | 8.0 |
| Mat. education: none | 7.7 | 7.7 | 6.1 | 9.1 | 6.6 | 7.9 | 7.2 | 4.9 | 5.5 | 4.0 | 6.0 | 11.1 |
| Mat. education: (some) prim. | 6.2 | 6.8 | 5.3 | 6.4 | 9.4 | 4.9 | 5.0 | 5.2 | 5.8 | 4.3 | 6.8 | 4.5 |
| Mother illiterate | 7.9 | 8.7 | 7.6 | 10.2 | 6.8 | 6.6 | 8.0 | 6.3 | 7.6 | 4.9 | 7.6 | 10.8 |
| Mother literate | 6.1 | 6.0 | 4.3 | 5.5 | 9.0 | 6.4 | 4.5 | 4.3 | 4.5 | 3.7 | 5.5 | 4.9 |
| Head education: none | 6.5 | 7.2 | 3.1 | 7.5 | 10.7 | 5.3 | 7.3 | 5.8 | 5.4 | 5.4 | 6.8 | 9.9 |
| Head education: (some) prim. | 7.3 | 7.3 | 7.4 | 7.9 | 6.2 | 7.3 | 5.3 | 4.7 | 5.8 | 3.5 | 6.2 | 6.5 |
| Assets: below median | 7.1 | 7.8 | 5.0 | 8.3 | 9.2 | 5.7 | 7.5 | 6.2 | 8.5 | 4.5 | 7.7 | 9.3 |
| Assets: above median | 6.9 | 6.4 | 6.5 | 6.6 | 5.7 | 8.1 | 4.7 | 4.2 | 3.8 | 3.9 | 5.5 | 5.9 |
| Child age: 0 to 5.9 mo | 4.3 | 5.0 | 2.9 | 6.1 | 5.4 | 2.8 | 3.4 | 3.0 | 3.3 | 2.8 | 3.3 | 4.5 |
| Child age: 6 to 11.9 mo | 8.7 | 8.8 | 7.3 | 9.1 | 9.7 | 8.4 | 6.9 | 5.5 | 4.9 | 6.1 | 4.9 | 9.8 |
| Child age: 12 to 17.9 mo | 9.0 | 8.9 | 8.9 | 9.6 | 7.6 | 9.0 | 7.6 | 6.3 | 9.7 | 2.8 | 9.4 | 10.1 |
| Child age: 18 to 23.9 mo | 5.9 | 6.0 | 4.3 | 5.8 | 8.7 | 5.7 | 5.7 | 5.4 | 4.1 | 4.9 | 8.0 | 6.3 |
| Child Weight-for-Length Z-score |  |  |  |  |  |  |  |  |  |  |  |  |
| All | -0.3±1.2 | -0.3±1.2 | -0.3±1.2 | -0.3±1.2 | -0.3±1.2 | -0.3±1.2 | -0.2±1.2 | -0.2±1.1 | -0.2±1.1 | -0.1±1.2 | -0.3±1.1 | -0.3±1.2 |
| Mat. education: none | -0.4±1.2 | -0.3±1.2 | -0.3±1.2 | -0.3±1.2 | -0.4±1.1 | -0.4±1.2 | -0.3±1.2 | -0.2±1.1 | -0.1±1.1 | -0.2±1.1 | -0.3±1.1 | -0.5±1.3 |
| Mat. education: (some) prim. | -0.3±1.2 | -0.3±1.2 | -0.4±1.2 | -0.3±1.2 | -0.2±1.3 | -0.2±1.1 | -0.2±1.2 | -0.2±1.2 | -0.3±1.1 | -0.1±1.2 | -0.2±1.1 | -0.1±1.2 |
| Mother illiterate | -0.3±1.2 | -0.4±1.2 | -0.3±1.1 | -0.3±1.1 | -0.5±1.2 | -0.3±1.2 | -0.3±1.2 | -0.3±1.2 | -0.3±1.1 | -0.3±1.2 | -0.2±1.1 | -0.5±1.2 |
| Mother literate | -0.3±1.2 | -0.3±1.2 | -0.4±1.2 | -0.3±1.3 | -0.2±1.2 | -0.3±1.2 | -0.2±1.2 | -0.1±1.1 | -0.2±1.1 | 0.0±1.1 | -0.3±1.1 | -0.2±1.3 |
| Head education: none | -0.4±1.2 | -0.4±1.2 | -0.4±1.3 | -0.4±1.2 | -0.3±1.1 | -0.3±1.2 | -0.3±1.2 | -0.2±1.2 | -0.3±1.1 | -0.1±1.2 | -0.4±1.2 | -0.5±1.2 |
| Head education: (some) prim. | -0.3±1.2 | -0.3±1.2 | -0.3±1.1 | -0.3±1.2 | -0.3±1.3 | -0.2±1.1 | -0.1±1.2 | -0.1±1.1 | -0.1±1.1 | -0.1±1.2 | -0.2±1.1 | -0.1±1.2 |
| Assets: below median | -0.3±1.2 | -0.3±1.2 | -0.3±1.0 | -0.3±1.2 | -0.4±1.2 | -0.3±1.2 | -0.4±1.2 | -0.3±1.2 | -0.4±1.2 | -0.2±1.1 | -0.4±1.2 | -0.5±1.2 |
| Assets: above median | -0.3±1.2 | -0.3±1.2 | -0.4±1.3 | -0.3±1.2 | -0.2±1.2 | -0.3±1.2 | -0.1±1.2 | -0.1±1.1 | -0.1±1.1 | -0.1±1.2 | -0.2±1.1 | -0.2±1.3 |
| Child age: 0 to 5.9 mo | 0.2±1.3 | 0.2±1.4 | 0.3±1.3 | 0.3±1.4 | 0.0±1.3 | 0.1±1.2 | 0.4±1.2 | 0.4±1.2 | 0.4±1.1 | 0.4±1.2 | 0.4±1.2 | 0.3±1.3 |
| Child age: 6 to 11.9 mo | -0.5±1.1 | -0.5±1.1 | -0.4±1.1 | -0.6±1.1 | -0.5±1.2 | -0.4±1.2 | -0.3±1.2 | -0.2±1.1 | -0.2±1.1 | -0.2±1.2 | -0.3±1.0 | -0.4±1.3 |
| Child age: 12 to 17.9 mo | -0.5±1.1 | -0.5±1.1 | -0.7±1.0 | -0.5±1.1 | -0.4±1.0 | -0.6±1.1 | -0.5±1.1 | -0.4±1.0 | -0.5±1.1 | -0.3±1.0 | -0.6±1.1 | -0.7±1.2 |
| Child age: 18 to 23.9 mo | -0.3±1.1 | -0.4±1.1 | -0.5±1.0 | -0.3±1.0 | -0.3±1.2 | -0.3±1.1 | -0.4±1.1 | -0.4±1.0 | -0.4±1.0 | -0.4±1.1 | -0.5±1.0 | -0.3±1.1 |

1 Data are mean ± SD unless otherwise specified.

**Supplemental Table 5:** Regression model to estimate the impact on wasting in children 0 to 23.9 mo in Burundi by maternal education and literacy, education of the head of household, household assets, and child age

|  | **Maternal education** | | **Maternal literacy** | | **Head education** | | **Assets** | | **Age** | | | |
| --- | --- | --- | --- | --- | --- | --- | --- | --- | --- | --- | --- | --- |
| **VARIABLES** | **None** | **(Some) primary** | **Not literate** | **Literate** | **None** | **(Some) primary** | **Below median** | **Above median** | **0 to 5.9 mo** | **6 to 11.9 mo** | **12 to 17.9 mo** | **18 to 23.9 mo** |
|  |  |  |  |  |  |  |  |  |  |  |  |  |
| Wave | 2.49 | -0.31 | 4.21** | -1.42 | 4.50*** | -1.14 | 3.13* | -2.88 | 2.12 | 1.22 | -0.37 | 1.48 |
|  | (1.72) | (2.05) | (1.92) | (1.50) | (1.17) | (2.02) | (1.76) | (2.40) | (2.55) | (2.91) | (3.97) | (1.82) |
| Wave x … |  |  |  |  |  |  |  |  |  |  |  |  |
| All T combined | -5.87*** | -1.20 | -6.25*** | -0.25 | -5.60*** | -1.62 | -4.78** | 0.37 | -4.15* | -5.43* | -2.75 | -1.92 |
|  | (2.19) | (2.19) | (2.41) | (1.63) | (2.03) | (2.18) | (2.15) | (2.77) | (2.84) | (3.62) | (4.44) | (2.41) |
| Child |  |  |  |  |  |  |  |  |  |  |  |  |
| Age, mo | 1.26*** | 0.96*** | 1.12*** | 1.17*** | 0.97*** | 1.18*** | 0.99*** | 1.37*** | -2.13 | 6.69 | -11.85 | 0.59 |
|  | (0.34) | (0.31) | (0.36) | (0.28) | (0.36) | (0.29) | (0.32) | (0.34) | (1.77) | (5.49) | (10.34) | (9.38) |
| Age^2^, mo^2^ | -0.05*** | -0.04*** | -0.04*** | -0.05*** | -0.04** | -0.05*** | -0.04*** | -0.06*** | 0.32 | -0.33 | 0.38 | -0.03 |
|  | (0.01) | (0.01) | (0.01) | (0.01) | (0.01) | (0.01) | (0.01) | (0.01) | (0.27) | (0.31) | (0.35) | (0.22) |
| Male | 4.05*** | 2.23* | 3.00*** | 3.16*** | 4.11*** | 2.23** | 3.57*** | 2.84*** | 1.28 | 2.96* | 3.91** | 4.11*** |
|  | (0.78) | (1.11) | (1.00) | (0.94) | (1.23) | (1.10) | (1.01) | (0.88) | (1.13) | (1.63) | (1.59) | (1.38) |
| Mother |  |  |  |  |  |  |  |  |  |  |  |  |
| Age, y | 0.05 | 0.15* | 0.24*** | -0.04 | 0.13 | 0.08 | 0.18** | -0.01 | 0.05 | 0.28** | 0.00 | 0.07 |
|  | (0.07) | (0.08) | (0.08) | (0.08) | (0.09) | (0.07) | (0.09) | (0.07) | (0.09) | (0.12) | (0.13) | (0.09) |
| Biological mother | -171.17 | -1,252.45*** | -277.64 | -1,090.20*** | 31.07 | -925.21*** | 454.99 | -764.74** | 8.60** | 227.71*** | 1,633.00** | -935.04*** |
|  | (372.24) | (153.51) | (411.75) | (196.78) | (132.83) | (284.10) | (544.11) | (331.60) | (3.30) | (40.88) | (769.20) | (230.40) |
| Height, cm | -1.16 | -7.80*** | -1.65 | -6.91*** | -0.02 | -5.58*** | 2.82 | -4.67** | 0.17 | 1.37*** | 10.18** | -5.85*** |
|  | (2.32) | (0.98) | (2.58) | (1.25) | (0.83) | (1.81) | (3.49) | (2.06) | (0.11) | (0.22) | (4.91) | (1.42) |
| Biological mother x height | 0.97 | 7.87*** | 1.66 | 6.84*** | -0.23 | 5.70*** | -2.96 | 4.76** |  | -1.36*** | -10.34** | 5.78*** |
|  | (2.32) | (0.98) | (2.58) | (1.24) | (0.83) | (1.81) | (3.48) | (2.04) |  | (0.26) | (4.89) | (1.41) |
| Education |  |  |  |  |  |  |  |  |  |  |  |  |
| Primary incomplete |  | -2.15 | -6.41* | -2.49 | -2.80 | -1.88 | -5.02** | -2.36 | 0.79 | -3.19** | 0.93 | -3.44*** |
|  |  | (1.41) | (3.79) | (1.63) | (3.11) | (1.77) | (2.50) | (1.75) | (1.44) | (1.42) | (1.52) | (1.27) |
| Primary complete |  |  | 0.19 | -1.09 | -2.81** | -0.33 | -0.85 | -1.22 | -4.60** | -3.09 | 3.08 | -4.74*** |
|  |  |  | (1.37) | (1.11) | (1.08) | (1.05) | (0.94) | (1.22) | (2.02) | (2.04) | (4.05) | (1.76) |
| Head of household education |  |  |  |  |  |  |  |  |  |  |  |  |
| Primary incomplete | -0.49 | 1.62 | -0.43 | 1.54 |  |  | 0.23 | 1.19 | -1.49 | 3.32* | 1.29 | -0.35 |
|  | (1.13) | (1.14) | (1.06) | (1.24) |  |  | (1.19) | (1.16) | (1.19) | (1.74) | (1.37) | (1.27) |
| Primary complete | -3.66** | -0.38 | -4.96** | -0.27 |  | -2.78* | -3.00 | -1.06 | 0.41 | -1.37 | -2.96 | -2.10 |
|  | (1.62) | (1.66) | (2.20) | (1.55) |  | (1.41) | (2.31) | (1.65) | (3.40) | (2.90) | (2.31) | (2.29) |
| Household |  |  |  |  |  |  |  |  |  |  |  |  |
| Dependency ratio |  |  |  |  |  |  |  |  |  |  |  |  |
| Tertile 2 | -0.20 | -0.20 | -1.56 | 1.36 | -0.68 | 0.16 | -1.62 | 1.40 | -1.04 | -0.20 | 2.67 | -1.65 |
|  | (1.15) | (1.24) | (1.40) | (1.16) | (1.47) | (1.03) | (1.40) | (1.07) | (1.41) | (1.63) | (2.11) | (1.66) |
| Tertile 3 | 2.01 | -1.74 | -0.50 | 1.46 | 1.85 | -0.49 | 2.37 | -1.60 | 1.38 | -1.17 | 0.63 | 0.02 |
|  | (2.12) | (1.41) | (2.18) | (1.24) | (1.92) | (1.34) | (1.89) | (1.21) | (2.18) | (1.91) | (2.21) | (1.94) |
| Assets above median | 0.05 | -1.19 | -1.46 | -0.02 | -1.55 | -0.26 |  |  | -2.75** | 1.82 | -1.01 | -1.48 |
|  | (1.14) | (0.99) | (1.27) | (0.83) | (1.19) | (1.01) |  |  | (1.36) | (1.78) | (1.71) | (1.02) |
| Constant | 197.60 | 1,237.68*** | 270.20 | 1,101.02*** | 4.33 | 907.17*** | -437.95 | 750.87** | -28.43 | -263.32*** | -1,510.90* | 949.52*** |
|  | (372.21) | (151.36) | (413.50) | (195.30) | (134.10) | (283.12) | (546.73) | (333.94) | (18.06) | (43.98) | (785.10) | (253.93) |
|  |  |  |  |  |  |  |  |  |  |  |  |  |
| Observations | 2,492 | 2,589 | 2,341 | 2,740 | 1,983 | 3,098 | 2,821 | 2,260 | 1,092 | 1,293 | 1,307 | 1,389 |
| R-squared | 0.02 | 0.02 | 0.02 | 0.02 | 0.02 | 0.02 | 0.02 | 0.03 | 0.02 | 0.02 | 0.02 | 0.04 |
| Number of collid | 60 | 60 | 60 | 60 | 60 | 60 | 60 | 60 | 59 | 60 | 60 | 60 |

Robust standard errors in parentheses; *** p<0.01, ** p<0.05, * p<0.1; one-sided tests for impact estimates

**Supplemental Table 6:** Regression model to estimate the impact on weight-for-length z-score in children 0 to 23.9 mo in Burundi by maternal education and literacy, education of the head of household, household assets, and child age.

|  | **Maternal education** | | **Maternal literacy** | | **Head education** | | **Assets** | | **Age** | | | |
| --- | --- | --- | --- | --- | --- | --- | --- | --- | --- | --- | --- | --- |
| **VARIABLES** | **None** | **(Some) primary** | **Not literate** | **Literate** | **None** | **(Some) primary** | **Below median** | **Above median** | **0 to 5.9 mo** | **6 to 11.9 mo** | **12 to 17.9 mo** | **18 to 23.9 mo** |
|  |  |  |  |  |  |  |  |  |  |  |  |  |
| Wave | -0.12* | 0.05 | -0.18** | 0.11 | -0.24*** | 0.11 | -0.10 | 0.12 | 0.12 | -0.00 | -0.03 | -0.06 |
|  | (0.06) | (0.10) | (0.07) | (0.09) | (0.09) | (0.08) | (0.08) | (0.13) | (0.21) | (0.11) | (0.11) | (0.12) |
| Wave x … |  |  |  |  |  |  |  |  |  |  |  |  |
| All T combined | 0.27*** | 0.06 | 0.31*** | -0.01 | 0.31*** | 0.03 | 0.16** | 0.07 | -0.03 | 0.27** | 0.17 | 0.01 |
|  | (0.07) | (0.11) | (0.10) | (0.09) | (0.11) | (0.09) | (0.09) | (0.14) | (0.23) | (0.14) | (0.13) | (0.14) |
| Child |  |  |  |  |  |  |  |  |  |  |  |  |
| Age, mo | -0.16*** | -0.17*** | -0.17*** | -0.16*** | -0.14*** | -0.18*** | -0.16*** | -0.18*** | 0.17** | -0.61** | 0.53 | -0.56 |
|  | (0.02) | (0.02) | (0.02) | (0.01) | (0.02) | (0.01) | (0.01) | (0.02) | (0.08) | (0.25) | (0.42) | (0.38) |
| Age^2^, mo^2^ | 0.01*** | 0.01*** | 0.01*** | 0.01*** | 0.00*** | 0.01*** | 0.01*** | 0.01*** | -0.04*** | 0.03** | -0.02 | 0.01 |
|  | (0.00) | (0.00) | (0.00) | (0.00) | (0.00) | (0.00) | (0.00) | (0.00) | (0.01) | (0.01) | (0.01) | (0.01) |
| Male | -0.15*** | -0.10** | -0.13** | -0.11** | -0.17** | -0.09* | -0.18*** | -0.06 | 0.00 | -0.10 | -0.21*** | -0.20*** |
|  | (0.05) | (0.05) | (0.05) | (0.05) | (0.07) | (0.05) | (0.05) | (0.04) | (0.09) | (0.08) | (0.06) | (0.06) |
| Mother |  |  |  |  |  |  |  |  |  |  |  |  |
| Age, y | -0.02*** | -0.01*** | -0.02*** | -0.01*** | -0.01*** | -0.02*** | -0.02*** | -0.01*** | -0.02*** | -0.01*** | -0.01** | -0.01* |
|  | (0.00) | (0.00) | (0.00) | (0.00) | (0.00) | (0.00) | (0.00) | (0.00) | (0.01) | (0.00) | (0.01) | (0.00) |
| Biological mother | 14.32 | 34.45*** | 17.08* | 28.39*** | 14.87* | 25.18** | -24.24 | 32.74*** | -1.21*** | -9.79*** | -23.00** | 28.94*** |
|  | (10.26) | (8.07) | (10.02) | (10.06) | (8.46) | (9.51) | (15.86) | (8.67) | (0.18) | (1.98) | (9.39) | (9.95) |
| Height, cm | 0.10 | 0.21*** | 0.11* | 0.17*** | 0.10* | 0.15** | -0.15 | 0.20*** | -0.02*** | -0.07*** | -0.14** | 0.19*** |
|  | (0.06) | (0.05) | (0.06) | (0.06) | (0.05) | (0.06) | (0.10) | (0.06) | (0.01) | (0.01) | (0.06) | (0.06) |
| Biological mother x height | -0.09 | -0.21*** | -0.11* | -0.17*** | -0.09* | -0.15** | 0.15 | -0.20*** |  | 0.07*** | 0.15** | -0.18*** |
|  | (0.06) | (0.05) | (0.06) | (0.06) | (0.05) | (0.06) | (0.10) | (0.05) |  | (0.01) | (0.06) | (0.06) |
| Education |  |  |  |  |  |  |  |  |  |  |  |  |
| Primary incomplete |  | 0.14* | 0.34 | 0.14 | 0.12 | 0.17* | 0.14 | 0.21* | 0.11 | 0.15** | -0.03 | 0.06 |
|  |  | (0.07) | (0.41) | (0.10) | (0.16) | (0.10) | (0.15) | (0.12) | (0.09) | (0.07) | (0.07) | (0.06) |
| Primary complete |  |  | -0.02 | 0.07 | 0.13** | 0.02 | 0.05 | 0.06 | 0.17 | 0.05 | 0.21 | 0.33 |
|  |  |  | (0.06) | (0.06) | (0.05) | (0.06) | (0.05) | (0.06) | (0.17) | (0.15) | (0.20) | (0.20) |
| Head of household education |  |  |  |  |  |  |  |  |  |  |  |  |
| Primary incomplete | 0.05 | -0.03 | 0.07 | -0.05 |  |  | 0.06 | -0.05 | 0.22** | -0.13* | -0.03 | -0.02 |
|  | (0.04) | (0.05) | (0.05) | (0.04) |  |  | (0.05) | (0.05) | (0.10) | (0.07) | (0.06) | (0.06) |
| Primary complete | 0.24* | 0.16* | 0.14 | 0.21** |  | 0.22*** | 0.31** | 0.14 | 0.32 | 0.17 | 0.32** | 0.01 |
|  | (0.13) | (0.09) | (0.14) | (0.09) |  | (0.08) | (0.14) | (0.10) | (0.27) | (0.15) | (0.13) | (0.12) |
| Household |  |  |  |  |  |  |  |  |  |  |  |  |
| Dependency ratio |  |  |  |  |  |  |  |  |  |  |  |  |
| Tertile 2 | -0.03 | -0.06 | -0.03 | -0.08 | 0.00 | -0.08 | -0.02 | -0.07 | 0.17 | -0.11 | -0.07 | -0.09 |
|  | (0.05) | (0.06) | (0.05) | (0.07) | (0.07) | (0.05) | (0.05) | (0.06) | (0.12) | (0.08) | (0.08) | (0.08) |
| Tertile 3 | 0.05 | 0.04 | 0.03 | 0.02 | 0.09 | -0.02 | -0.09 | 0.13* | 0.25* | -0.02 | -0.00 | -0.04 |
|  | (0.08) | (0.08) | (0.08) | (0.08) | (0.07) | (0.08) | (0.07) | (0.08) | (0.13) | (0.09) | (0.08) | (0.10) |
| Assets above median | 0.08 | 0.12*** | 0.13** | 0.08** | 0.18*** | 0.07 |  |  | 0.12 | 0.15** | 0.04 | 0.11* |
|  | (0.06) | (0.04) | (0.06) | (0.04) | (0.06) | (0.04) |  |  | (0.10) | (0.07) | (0.06) | (0.06) |
| Constant | -14.37 | -32.74*** | -16.02 | -27.52*** | -14.90* | -23.41** | 24.64 | -31.22*** | 5.46*** | 13.36*** | 17.21* | -25.52** |
|  | (10.29) | (8.12) | (10.09) | (10.07) | (8.37) | (9.42) | (15.92) | (8.94) | (1.35) | (2.32) | (9.20) | (10.69) |
|  |  |  |  |  |  |  |  |  |  |  |  |  |
| Observations | 2,492 | 2,589 | 2,341 | 2,740 | 1,983 | 3,098 | 2,821 | 2,260 | 1,092 | 1,293 | 1,307 | 1,389 |
| R-squared | 0.08 | 0.09 | 0.09 | 0.09 | 0.07 | 0.10 | 0.08 | 0.10 | 0.06 | 0.06 | 0.04 | 0.03 |
| Number of collid | 60 | 60 | 60 | 60 | 60 | 60 | 60 | 60 | 59 | 60 | 60 | 60 |

Robust standard errors in parentheses; *** p<0.01, ** p<0.05, * p<0.1; one-sided tests for impact estimates

1. Abbreviations used: BCC: behavior change communication; CSB: corn-soy blend; HAZ: height-for-age Z-score; NGO: non-governmental organization; THP: *Tubaramure* health promotor; [↑](#footnote-ref-1)
